# B lymphocytes and tertiary lymphoid structures have a prognostic impact on penile squamous cell carcinoma

**DOI:** 10.1002/2056-4538.70059

**Published:** 2025-11-14

**Authors:** Nicolette Zavillová, Petr Waldauf, Michaela Kendall Bártů, David Čapka, Jan Hojný, Zuzana Prouzová, Radoslav Matěj, Roman Zachoval, Jan Hrudka

**Affiliations:** ^1^ Department of Urology, 3rd Faculty of Medicine of Charles University Thomayer University Hospital Prague Czech Republic; ^2^ Department of Anesthesia and Intensive Care Medicine, 3rd Faculty of Medicine, Charles University University Hospital Kralovske Vinohrady Prague Czech Republic; ^3^ Department of Pathology, 1st Faculty of Medicine, Charles University General University Hospital Prague Czech Republic; ^4^ Department of Urology, 3rd Faculty of Medicine, Charles University University Hospital Kralovske Vinohrady Prague Czech Republic; ^5^ Department of Pathology, 3rd Faculty of Medicine, Charles University University Hospital Kralovske Vinohrady Prague Czech Republic; ^6^ Department of Pathology and Molecular Medicine, 3rd Faculty of Medicine, Charles University Thomayer University Hospital Prague Czech Republic

**Keywords:** penile, penis, tertiary lymphoid structures (TLSs), B‐cell immunoscore (B‐IS), penile squamous cell carcinoma (pSCC), prognosis, CD20, CD138, tumor‐infiltrating lymphocytes (TILs)

## Abstract

Several prognostic markers, including tumor‐infiltrating lymphocytes, which have been recently identified in penile squamous cell carcinoma (pSCC), have focused mostly on T cells. The prognostic role of B cells and tertiary lymphoid structures (TLSs) has not yet been sufficiently described. We examined whole tissue sections histopathologically for TLSs and immunohistochemically for CD20 and CD138. The B‐cell immunoscore (B‐IS) divided the cohort into five categories based on the expression of these two B‐cell/plasma cell markers (CD20 and CD138, respectively). Patients with fewer TLSs had worse overall survival (OS) [hazard ratio (HR) = 2.17; 95% CI: 0.94–5; *p* = 0.069]. A significant association was identified between a high TLS diameter and the presence of a lymphocytic infiltrate [odds ratio (OR) = 2.2442; 95% CI: 1.1022–4.55; *p* = 0.0208]. Patients with low B‐IS (HR = 1.89, 95% CI: 1.18–3.03, *p* = 0.008), a low number of CD20^+^ cells in the tumor center (HR = 1.67, 95% CI: 1.04–2.7, *p* = 0.035), and a low number of CD20^+^ cells at the tumor invasion front (HR = 1.69, 95% CI: 1.06–2.78; *p* = 0.028) had significantly worse OS. High B‐ISs were strongly associated with a mutated p53 profile detected by immunohistochemistry (OR = 4.76, 95% CI: 1.32–25, *p* = 0.011), low T‐cell immunoscores (OR = 0.49; 95% CI: 0.23–1.03; *p* = 0.051), and brisk lymphocytic infiltration (OR = 2.0417, 95% CI: 1.01–4.76; *p* = 0.037). High CD20^+^ cell counts at the invasion front were associated with histological grade 3 disease (OR = 2.44, 95% CI 1.15–5.26, *p* = 0.015). An association was also observed between low B‐IS and mutations in *KMT2D* (OR 0.31, 95% CI: 0.07–1.21, *p* = 0.057) and *EGFR* (OR = ∞, 95% CI: 0.86–∞, *p* = 0.053). In conclusion, high numbers of tumor‐infiltrating B cells within TLSs represent a favorable prognostic marker in pSCC. These findings emphasize the need to identify novel microscopic prognostic markers during pathological assessment to guide early and appropriate therapeutic strategies.

## Introduction

Penile cancer is a rare malignancy in developed countries, characterized by a highly variable prognosis and significant morbidity. Despite advances in medicine, its associated mortality has remained largely unchanged [1]. The current understanding underscores the importance of traditional histopathological markers such as tumor grade and TNM stage in prognostication and treatment decision‐making. However, frequent diagnosis at late stages [2] and the presence of occult lymph node metastases highlight the need for more refined prognostic tools. Early‐stage treatment aims to preserve the organ through conservative surgical excision, whereas advanced cases require multimodal approaches, including systemic oncological therapy [3]. Regional lymph node metastasis is a key independent predictor of poor OS – notably, approximately 25% of patients present with occult lymph node micrometastases undetectable by clinical examination. Consequently, there is a critical need to identify novel prognostic biomarkers to improve risk stratification and guide clinical management in this disease.

Emerging evidence supports the critical role of the tumor immune microenvironment in modulating cancer progression and therapeutic responsiveness. In our recent work, we demonstrated that the density of the peritumoral lymphocytic infiltrate, as well as the immunoscore based on tumor‐infiltrating T lymphocytes, provides significant prognostic information [4]. In our second study, reduced infiltration of CTLA4^+^ immune cells at the invasive margin of the tumor was identified as a prognostic factor associated with decreased overall and cancer‐specific survival [5]. These findings align with results from other tumor types, where immune‐related biomarkers are increasingly used to stratify patients and predict response to immunotherapy.

Despite these advances, several important aspects remain unresolved. In particular, the role of B lymphocytes and tertiary lymphoid structures (TLSs) in penile SCC is largely unexplored.

TLSs are ectopic lymphoid formations that arise at sites of chronic inflammation, including within the tumor microenvironment. They represent heterogeneous entities that range from loosely organized cellular aggregates (early or immature TLSs) to well‐structured formations resembling primary or secondary follicles (the latter referred to as secondary TLSs). Mature TLSs often harbor germinal centers and high endothelial venules. Their cellular composition includes stromal elements together with diverse innate and adaptive immune populations. Fully developed, follicle‐like TLSs contain germinal centers composed of proliferating germinal center B cells supported by follicular dendritic cells, surrounded by naïve or follicular B cells, and bordered by follicular helper T cells. In addition, TLSs are composed of T lymphocytes, plasma cells, mature dendritic cells, and macrophages.

TLS formation is triggered by persistent antigen exposure and local inflammatory cytokines. Functionally, TLSs provide a local site for antigen presentation, specific B‐cell‐mediated immune response, and influx of immune cells into the tumor microenvironment. The presence of TLSs has been associated with improved prognosis in various cancers, as it facilitates localized immune responses and may enhance the efficacy of immunotherapies [6, 7, 8].

The role of TLSs in penile cancer is an emerging area of research. B‐lymphocytes, as an integral part of the immune microenvironment, can positively and negatively regulate immune responses [9, 10]. Given their fundamental role in the architecture of TLSs, they contribute to the immune landscape of tumors. However, the functional role of the range of B‐cell subsets is not yet well understood [9].

Based on these observations, we aimed to investigate the prognostic significance of TLSs and B lymphocytes in pSCC.

## Materials and methods

### Patients and follow‐up

All histologically confirmed invasive pSCCs, obtained through surgical procedures (excision, circumcision, partial or total penectomy, and lymphadenectomy) in three involved institutions between 2000 and 2024, were included in the analysis.

Representative formalin‐fixed paraffin‐embedded (FFPE) tumor tissue blocks, along with all available hematoxylin–eosin‐stained slides, were retrieved from the archives of the three pathology departments in the Czech Republic. All pathological stages were considered, irrespective of neoadjuvant or adjuvant therapy.

Follow‐up information was collected from clinical records or extracted from the Czech National Oncological Registry. OS was calculated from the date of surgery to the date of death or censored at the last known follow‐up date. Tumor staging was assigned using medical documentation and according to the TNM Classification and the Union for International Cancer Control criteria.

### Histopathology evaluation

All hematoxylin and eosin (H&E)‐stained slides retrieved from the archives of participating pathology departments were independently evaluated by two experienced pathologists (JH, ZP), who were blinded to the patient follow‐up data. Tumor grading was assigned based on the criteria established by the International Society of Urological Pathology [11].

The presence or absence of lymphatic, vascular, and perineural invasion was systematically recorded. Tumor budding, intratumoral and peritumoral lymphocytic infiltration, and expression of p16, p53, and PD‐L1 were assessed. The detailed histopathological methods are described in our previous publications [4, 5]. Immunohistochemistry p53 profiles were classified as wild type pattern in case of heterogeneous nuclear staining in tumor cells. Mutated pattern was recorded, if a strong uniform nuclear immunohistochemical signal in ≥80% of tumor cells (overexpression) was present. Null phenotype was recorded, if the nuclei of tumor cells were completely negative. For statistical analysis, both mutated patterns were clustered together.

### Evaluation of TLSs

**Figure 1:**
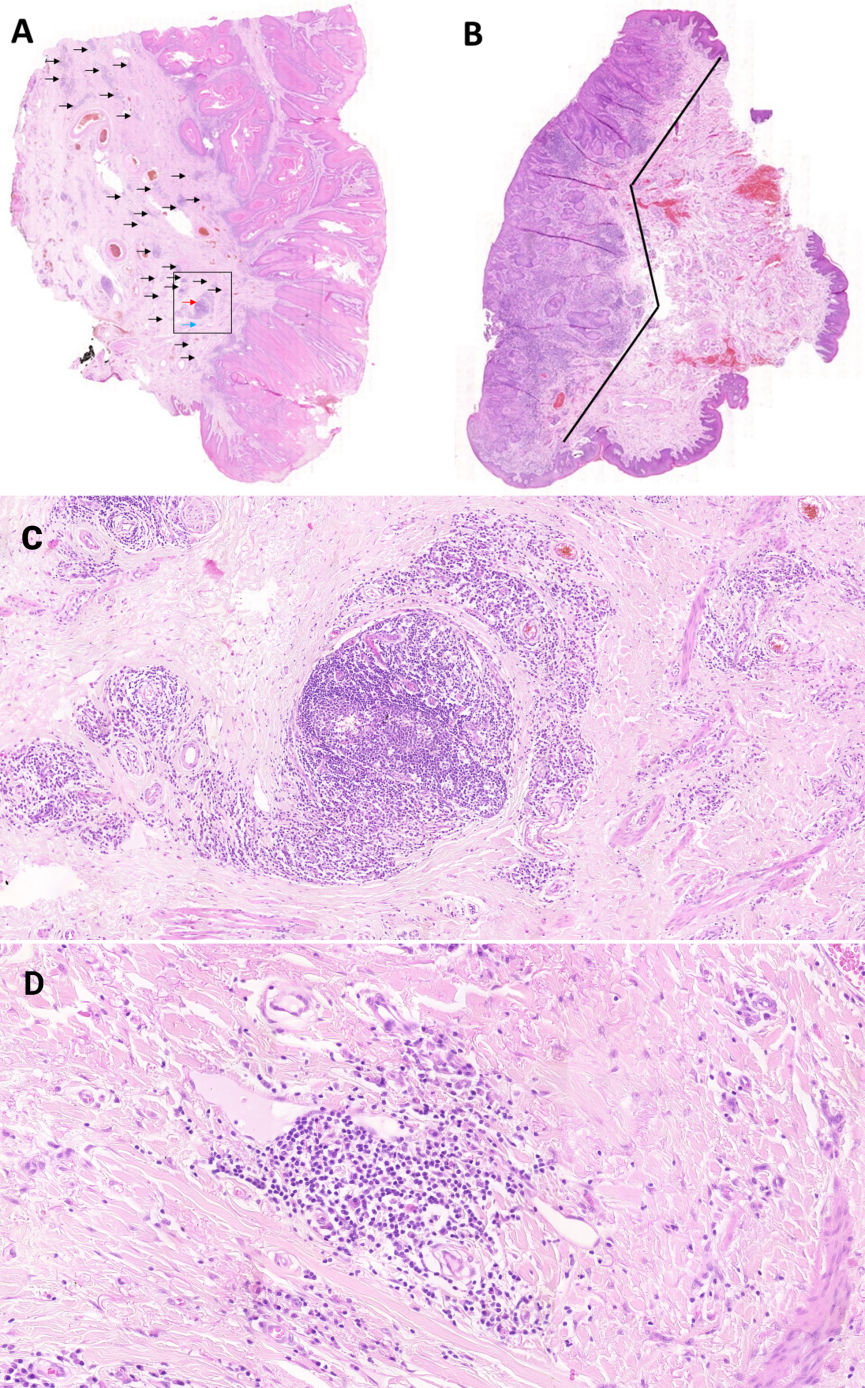
Histological slide scans of penile squamous cell carcinoma with TLSs. (A) The histological section demonstrates multiple TLSs, indicated by black arrows. The red arrow highlights a secondary TLS; the blue arrow highlights a primary TLS. The outlined square represents a 5‐mm^2^ area used for TLS quantification. (B) Histological section showing the absence of TLSs. The solid line delineates the length of the invasive tumor front. (C) Higher magnification view of a secondary TLS displaying a well‐organized lymphoid follicle with a germinal center and surrounding immune cell populations. (D) Higher magnification view of a primary TLS.

H&E‐stained slides were scanned using the 3DHISTECH Panoramic Desk II DW scanner. To assess the presence of TLSs, lymphocytic aggregates were manually identified along the entire tumor invasion front using CaseViewer software (3DHISTECH). For TLS quantification, we adopted the method used by Karjula *et al* [12]. Diffuse lymphocytic infiltrate was not regarded as a TLS, which was reserved only for well‐demarcated clusters of lymphocytes measuring ≥150 μm. For quantitative analysis, the number of TLSs was normalized per millimeter of invasion front length. The peritumoral region was defined as the area extending up to 1,000 μm from the tumor nest boundary, in accordance with preliminary observations that indicated this as the exclusive site of TLS formation [13]. TLS density was subsequently standardized and expressed as the number of TLSs per five square millimeters of peritumoral area. Additionally, the maximum diameter of the largest TLS in the sample was measured, and the presence of secondary follicle formation within TLSs was documented (Figure 1).

### Immunohistochemistry

A total of 152 representative FFPE tumor tissue blocks were utilized for immunohistochemical analysis. Tissue sections of 4 μm thickness were processed using the Ventana BenchMark ULTRA automated staining platform (Ventana Medical Systems, Tucson, AZ, USA). Monoclonal antibodies targeting CD20 (clone L26, dilution 1:100, Dako, Denmark) and CD138 (clone B‐A38, ready to use, Cell Marque, Germany) were applied.

Immunoreactivity was visualized using the UltraView Universal DAB Detection System (Ventana Medical Systems), followed by hematoxylin counterstaining. The stained slides were subsequently dehydrated and mounted in xylene‐based mounting medium.

### Immunoscore

The quantification of TILs was performed on tissue sections immunostained with monoclonal antibodies against CD20 and CD138. Stained slides were digitized using the 3DHISTECH Panoramic Desk II DW scanner, and manual cell counts were conducted with CaseViewer software (3DHISTECH), reporting the number of positively stained lymphocytes per square millimeter of analyzed tissue.

For each marker, TIL density was assessed independently in two anatomically distinct regions: the tumor center and the tumor invasive front, using two representative high‐power fields per region. To stratify the cohort based on TIL number, an adapted version of the immunoscore (IS) methodology developed initially by Galon *et al* for colorectal carcinoma [14] was employed.

**Figure 2:**
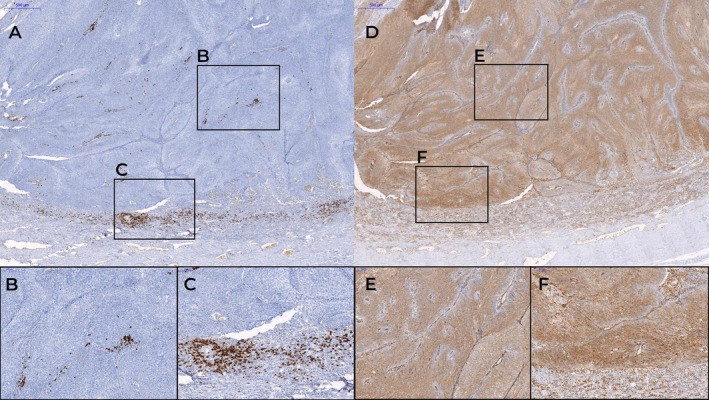
Immunohistochemical staining of tumor‐infiltrating B cells in penile squamous cell carcinoma using anti‐CD20 and anti‐CD138 antibodies. Tumor‐infiltrating lymphocytes were evaluated by quantifying CD20^+^ and CD138^+^ cells within 1 mm^2^ areas, both in the tumor center and at the invasive front, using CaseViewer software. The black rectangles indicate the regions of 1 mm^2^ selected for analysis. (A) Example of a high CD20^+^ immunoscore. (B) Higher magnification of high B‐IS in the CD20^+^ tumor center. (C) Higher magnification of high B‐IS in the CD20^+^ tumor invasion front. (D) Example of a high immunoscore CD138^+^. (E) Higher magnification of high B‐IS in the CD138^+^ tumor center. (F) Higher magnification of high B‐IS in the CD138^+^ tumor invasion front. This figure was created with CaseViewer software (3DHISTECH).

**Figure 3:**
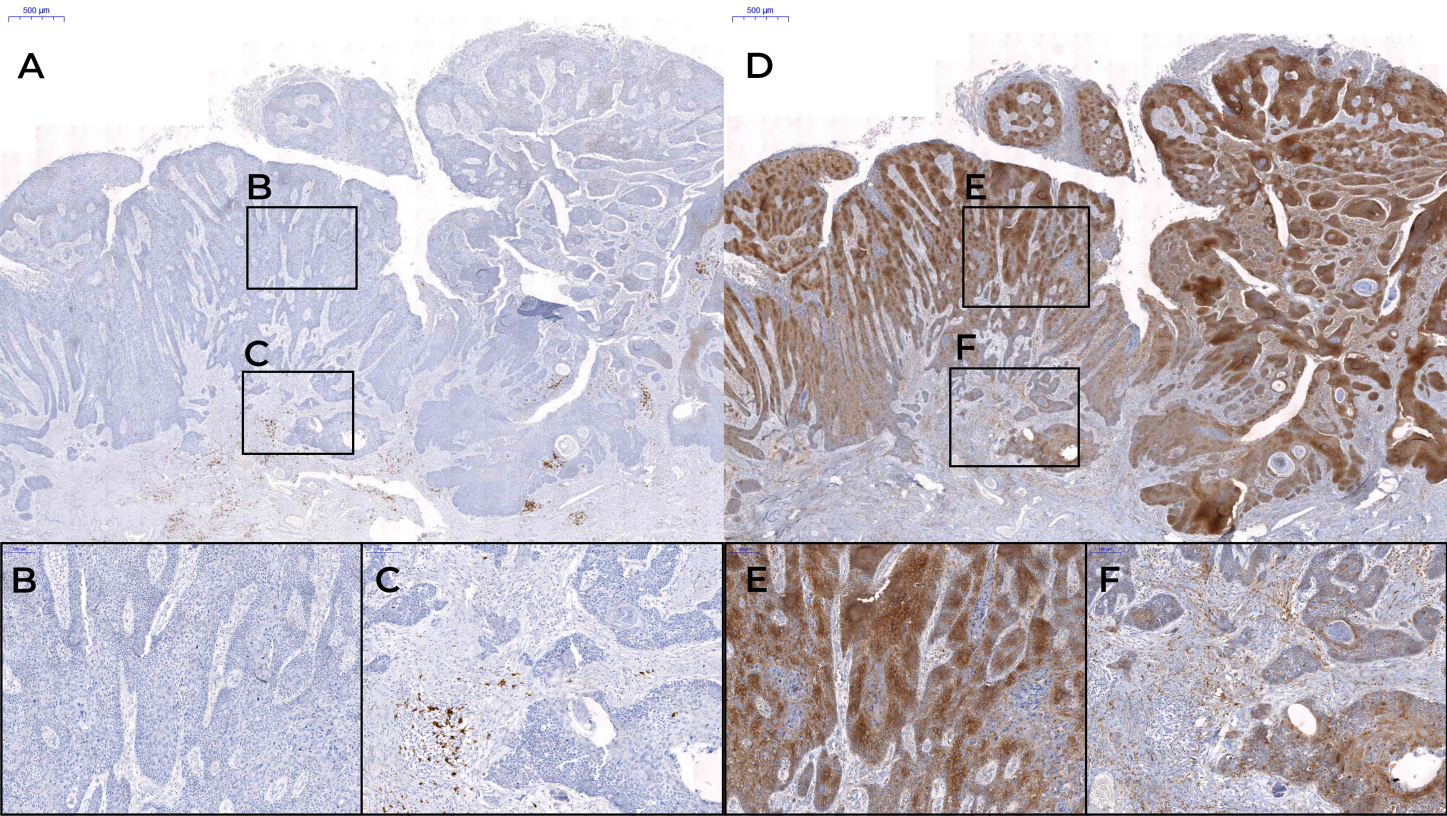
Immunohistochemical staining of tumor‐infiltrating B cells in penile squamous cell carcinoma using anti‐CD20 and anti‐CD138 antibodies. Tumor‐infiltrating lymphocytes were evaluated by quantifying CD20^+^ and CD138^+^ cells within 1 mm^2^ areas, both in the tumor center and at the invasive front, using CaseViewer software. The black rectangles indicate the regions of 1 mm^2^ selected for analysis. (A) Example of a low CD20^+^ immunoscore. (B) Higher magnification of low B‐IS in the CD20^+^ tumor center. (C) Higher magnification of low B‐IS in the CD20^+^ tumor invasion front. (D) Example of a low CD138^+^ immunoscore. (E) Higher magnification of low B‐IS in the CD138^+^ tumor center. (F) Higher magnification of low B‐IS in the CD138^+^ tumor invasion front. This figure was created with CaseViewer software (3DHISTECH).

The B‐cell immunoscore (B‐IS) was calculated by dichotomizing the expression levels of CD20^+^ and CD138^+^ TILs in both regions according to the median value for each variable, resulting in a binary classification (high – Figure 2 versus low – Figure 3) per site and marker. The combined scoring yielded five distinct IS categories, from IS 0 (low expression of all four parameters), IS 1 (three low expressions and one high expression of parameters), IS 2 (two low expressions and two high expressions of parameters), IS 3 (one low expression and three high expressions of parameters) to IS 4 (high expression across all four parameters), enabling integrated assessment of spatial and phenotypic immune infiltration.

### Next‐generation sequencing (NGS) analysis of DNA

NGS data were collected and analyzed in our previous publication where the methods are described in detail [15]. Alterations in *TP53*, *CDKN2A*, *ATM*, *EPHA7*, *POT1*, *CHEK1*, *GRIN2A*, and *EGFR* were found to be significantly associated with reduced OS [15]. In this study, we analyzed associations of the above‐mentioned variables (TLS, B cells, etc.) with gene alterations present in 10 or more cases. The agreement of p53 immunohistochemistry profile with *TP53* mutational status determined by NGS was assessed using Cohen's kappa coefficient (unpublished data).

### Statistical analysis

All analyses were conducted using R version 4.5.1 (2025‐06‐13) [16], with survival analyses executed through the survival package, version 3.4‐0. OS was analyzed using the Kaplan–Meier method, with statistical significance assessed via the log‐rank test. Confidence intervals (CIs) were estimated using the log–log transformation. In addition, restricted mean survival time (RMST) analysis was performed, with 95% CIs reported.

To estimate the hazard ratios (HRs) for individual parameters, univariate Cox proportional hazards regression models were employed, each with corresponding 95% CIs. Associations between categorical variables were assessed using Pearson's chi‐square test and interpreted as univariate logistic regression. All survival analyses were truncated at the 10‐year follow‐up. For both survival and chi‐square analyses, variables were analyzed in predefined categorical groupings as in our previous two studies [4, 5].

TLS variables (TLS number per mm of invasion front length, TLS density per five square millimeters, and maximal TLS diameter) were binarized according to the optimal cutoff value using the function surv_cutpoint (survminer) in R software. Secondary TLSs were binarized nominally (present/absent). The B‐IS was binarized arbitrarily as B‐IS 0–1/B‐IS 2–4. Additionally, we assessed the association between the DNA NGS data obtained from our previous study [15] and the current results.

In the subsequent analysis, multivariate Cox regression was utilized to evaluate OS, adjusting for variables that demonstrated statistically significant associations in the chi‐square test, as well as for the patient's age. A *p* value threshold of <0.05 defined statistical significance.

### Ethics statement

The study was approved by the institutional review board and the University Hospital Královské Vinohrady Ethics Committee, approval number EK‐VP1261012020. All the research was performed in accordance with the Declaration of Helsinki.

## Results

Univariate logistic regression/Pearson's chi‐square test for binarized variables is available in supplementary material, Table S1.

### TLSs

The mean number of TLSs was 0.7 per millimeter of invasion front length (range: 0–6.5; SD: 0.9). The mean TLS density was 4.1 per 5 mm^2^ (range: 0–18; SD: 4.4). The mean maximal diameter of TLSs was 0.3 mm (range: 0–1.8; SD: 0.3). Secondary TLSs were identified in 18 patients.

A total of 173 patients were enrolled in this study, with the median age at the time of surgery being 67.7 years (range: 31.4–91.8). The distribution of T stages and histological grades within the cohort is summarized in Table 1. The cohort comprised 99 p16+ and 72 p16− cases (p16 status could not be determined in two cases due to technical issues). Among these patients, 82 deaths were recorded during follow‐up. The RMST was 8.138 years, with a median survival of 6.004 years.

**Table 1:** Distribution of T stage and histological grade of our cohort

T stage	T1a	77
	T1b	25
	T2	41
	T3	24
	T4	2
	Tis	2
	Tx	2
Grade	G1	43
	G2	78
	G3	52

**Figure 4:**
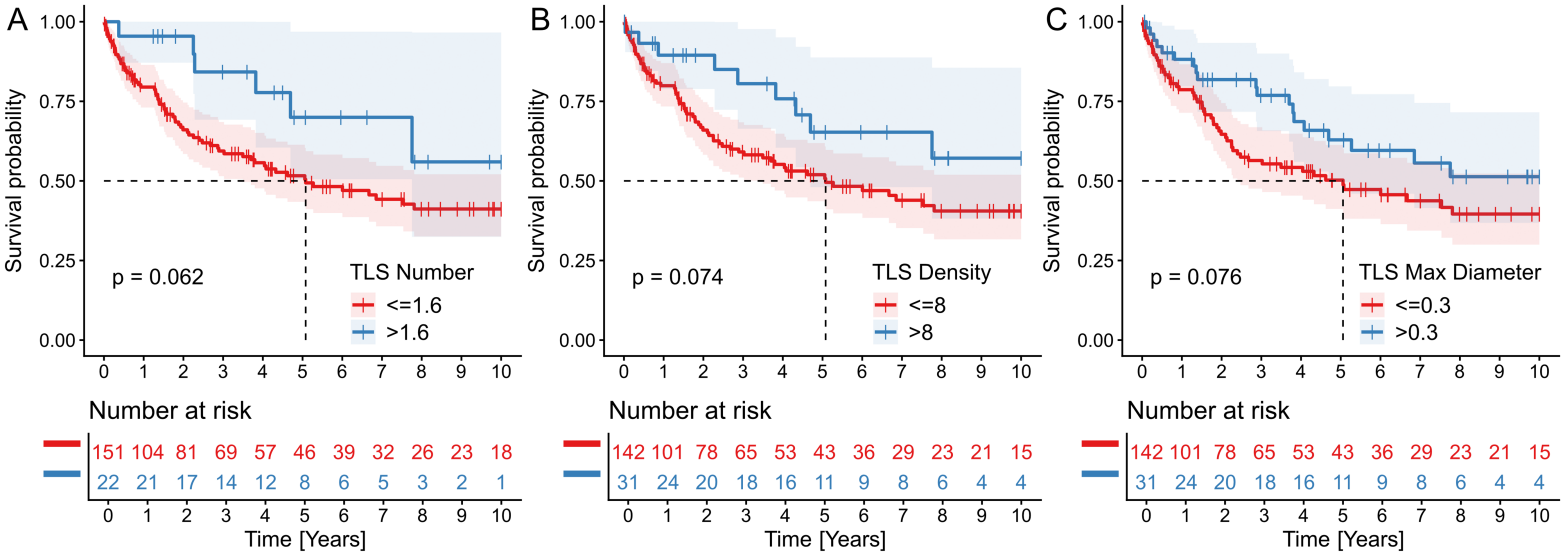
Kaplan–Meier curves illustrating overall survival based on TLS‐related parameters. (A) OS according to TLS number. (B) OS according to TLS density. (C) OS according to the maximal TLS diameter. Dashed vertical lines are drawn from the median survival time on the *x*‐axis to the Kaplan‐Meier curve. Dashed vertical lines on the *y*‐axis show 50% of the study cohort. This figure was created with R software version 4.5.1, package ggpod2 version 4.0.0.

A statistically borderline negative prognostic impact was observed for the low number of TLSs, with an optimal cutoff of 1.6 TLSs per millimeter of invasion front length [HR = 2.17; 95% CI: 0.94–5; *p* = 0.069; Kaplan–Meier (KM) *p* = 0,062; restricted mean survival (rmean) = 5,546 versus 7,567 years] (Figure 4A). Similarly, we observed nonsignificant trends towards worse prognosis for two additional TLS‐related parameters: low TLS density, with an optimal cutoff of 8 TLSs per 5 mm^2^ (HR = 1.85; 95% CI: 0.93–3.70; *p* = 0.079; KM *p* = 0,074; rmean = 5,521 versus 7,279 years) (Figure 4B), and low maximal TLS diameter, with a threshold of 0.5 mm (HR = 1.59; 95% CI: 0.95–2.70; *p* = 0.079; KM *p* = 0,076; rmean = 5,396 versus 6,749 years) (Figure 4C). The presence of secondary TLSs was not significantly associated with prognosis, possibly due to the small number of patients with secondary TLSs.

A significant association was identified between a reduced TLS diameter and non‐brisk/absent lymphocytic infiltrate (OR = 2.442; 95% CI: 1.22–5; *p* = 0.008). Nonsignificant associations were noted between lymphatic invasion and both a lower number of TLSs (OR = 1.96; 95% CI: 0.95–4.17; *p* = 0.06) and reduced TLS diameter (OR = 2.17; 95% CI: 0.94–5.26; *p* = 0.068). All research data are available in supplementary material, Table S2.

### CD20 and CD138

Concerning the immunoscore, the median number of B cells/mm^2^ was used (CD20 tumor center = 50, CD20 invasion front = 3,330, CD138 tumor center = 70, and CD138 invasion front = 200) to categorize the cohort into high/low cases, dividing the cases into five immunoscore subgroups as described earlier. In the survival analysis of particular CD20^+^ and CD138^+^ cell counts, the optimal cutpoint values rendered by the function surv_cutpoint (survminer) in R software were CD20 tumor center = 31, CD20 invasion front = 2,800, CD138 tumor center = 24, and CD138 invasion front = 680.

**Figure 5:**
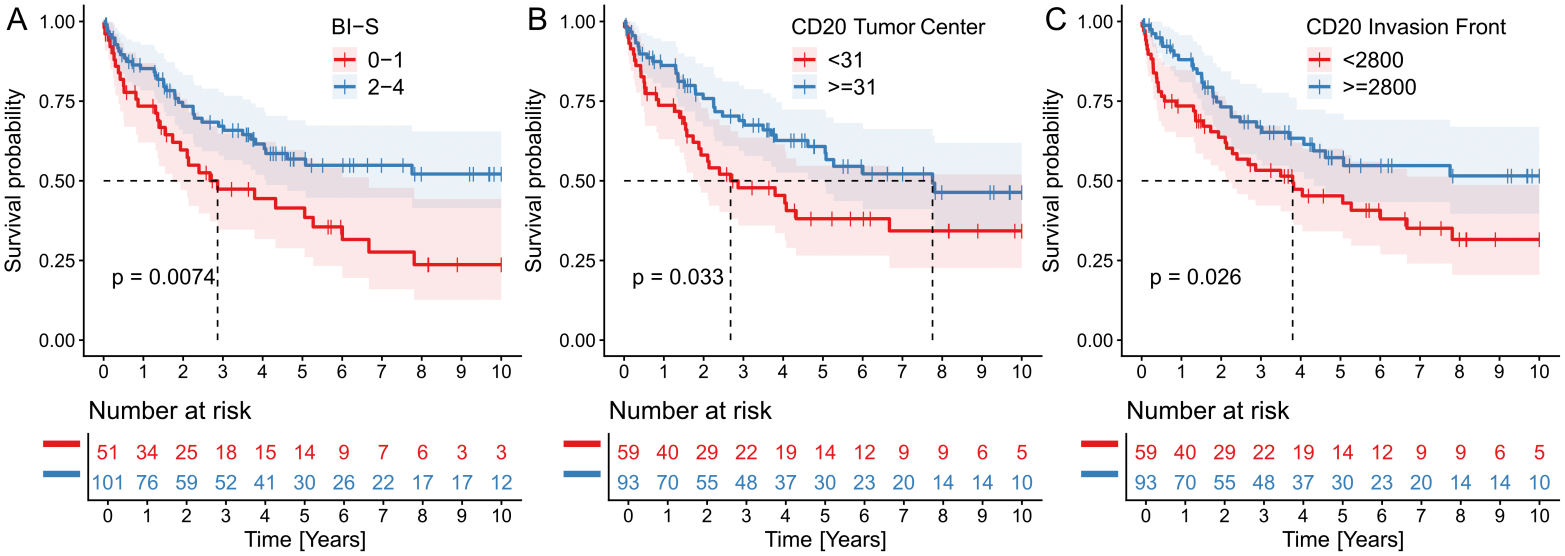
Kaplan–Meier curves illustrating overall survival based on B‐cell infiltration parameters. (A) OS according to B‐IS. (B) OS is determined by the expression of CD20^+^ cells in tumor centers. (C) OS is determined by the expression of the CD20^+^ cells at the invasion front. Dashed vertical lines are drawn from the median survival time on the *x*‐axis to the Kaplan‐Meier curve. These lines indicate the median survival corresponding to a 50% survival probability. This figure was created with R software version 4.5.1, package ggpod2 version 4.0.0.

The immunohistochemistry analysis was feasible in 152 of 173 patients. Twenty‐one patients were excluded due to technical limitations (i.e., insufficient amount of representative tumor tissue). During follow‐up 69 deaths were recorded. A low B‐IS 0–1, had a significant negative prognostic impact (HR = 1.89; 95% CI: 1.18–3.03; *p* = 0.008; KM *p* = 0,0074; rmean = 4,335 versus 6,320 years) (Figure 5A).

Low CD20 in the tumor center (HR = 1.67; 95% CI: 1.04–2.70; *p* = 0.035; KM *p* = 0,033; rmean = 4,675 versus 6,240 years) (Figure 5B) and on the invasion front (HR = 1.69; 95% CI: 1.06–2.78; *p* = 0.028; KM *p* = 0,026; rmean = 4,812 versus 6,370 years) (Figure 5C) had a significant negative prognostic impact, both of which were binarized at the optimal cutoff point. No significant impact on survival was observed according to the number of CD138.

For p53 immunohistochemistry versus *TP53* status by NGS (*n* = 145), Cohen's kappa reached *K* = 0.693 (*p* < 0.0001), which indicates substantial concordance between the two methods. There was a strong association between low B‐IS and p53 wild type status (OR = 4.76; 95% CI: 1.32–25; *p* = 0.011), low T‐cell immunoscore (OR = 0.49; 95% CI: 0.23–1.03; *p* = 0.051) and brisk lymphocytic infiltrate (OR = 2.17; 95% CI: 1.01–4.76; *p* = 0.037). No statistically significant associations of B‐cell variables with tumor mutational burden (TMB), PD‐L1 expression, or p16 expression were identified.

In the multivariate Cox proportional hazards analysis, patients with a B‐IS 2–4 demonstrated significantly improved OS compared with those with lower scores (HR = 0.39; 95% CI: 0.23–0.65; *p* < 0.001) when adjusted for p53 status (mutated versus wild type based on immunohistochemistry). Patients with a non‐brisk or absent lymphocytic infiltrate, when adjusted for the B‐IS, had significantly worse OS (HR = 2.62; 95% CI: 1.49–4.60; *p* < 0.001). In the multivariate analysis adjusted for the T‐cell immunoscore, a B‐IS 2–4 was associated with a trend towards improved OS, although this difference did not reach statistical significance (HR = 0.65; 95% CI: 0.40–1.06; *p* = 0.084). The results are summarized in Table 2 and all research data are available in supplementary material, Table S3.

**Table 2:** Overall survival analysis censored at 10 years with documented parameters

10 years analysis	Cutoff	*n*	Deaths	rmean	Median	*p* *	HR Cox regression (95% CI)	*p* ^†^	HR Cox adjusted on age (95% CI)	*p* ^‡^
TLS number	≤1.6	151	70	5.546	5.079	**0.062**	0.46 (0.20–1.06)	**0.069**	0.71 (0.3–1.68)	0.4
	>1.6	22	22	7.567	NA					
TLS density	≤8	142	67	5.521	5.079	**0.074**	0.54 (0.27–1.07)	**0.079**	0.73 (0.36–1.49)	0.4
	>8	31	9	7.279	NA					
Maximal TLS diameter	≤0.5	120	57	5.396	5.057	**0.076**	0.63 (0.37–1.05)	**0.079**	0.9 (0.53–1.55)	0.7
	>0.5	53	19	6.749	NA					
Secondary TLSs	Yes	18	6	6.718	7.756	0.34	0.67 (0.29–1.54)	0.3	0.94 (0.4–2.19)	0.9
	No	155	70	5.696	5.27					
B Immunoscore	0 + 1	51	31	4.335	2.866	**0.0074**	0.53 (0.33–0.85)	**0.008**	0.65 (0.4–1.05)	0.081
	2–4	101	38	6.32	NA					
CD20 Tumor center	<31	59	33	4.675	2.678	**0.033**	0.6 (0.37–0.96)	**0.035**	0.55 (0.34–0.89)	**0.015**
	≥31	93	36	6.240	7.756					
CD20 Invasion front	<2,800	69	39	4.812	3.797	**0.026**	0.59 (0.36–0.94)	**0.028**	0.63 (0.39–1.02)	0.062
	≥2,800	83	30	6.37	NA					
CD138 Tumor center	<24	42	23	4.813	3.797	0.18	0.71 (0.43–1.17)	0.2	0.67 (0.4–1.11)	0.12
	≥24	110	46	5.931	6.004					
CD138 Invasion front	<680	120	57	5.38	5.057	0.2	0.67 (0.36–1.25)	0.2	0.56 (0.3–1.06)	0.075
	≥680	32	12	6.457	3.496					

Significant *p* values are in bold.

CI, confidence interval; HR, hazard ratio; NA, not applicable; rmean, restricted mean survival time.

* Kaplan–Meier.

^†^ Cox regression.

^‡^ Age adjusted.

### Associations with NGS data

**Figure 6:**
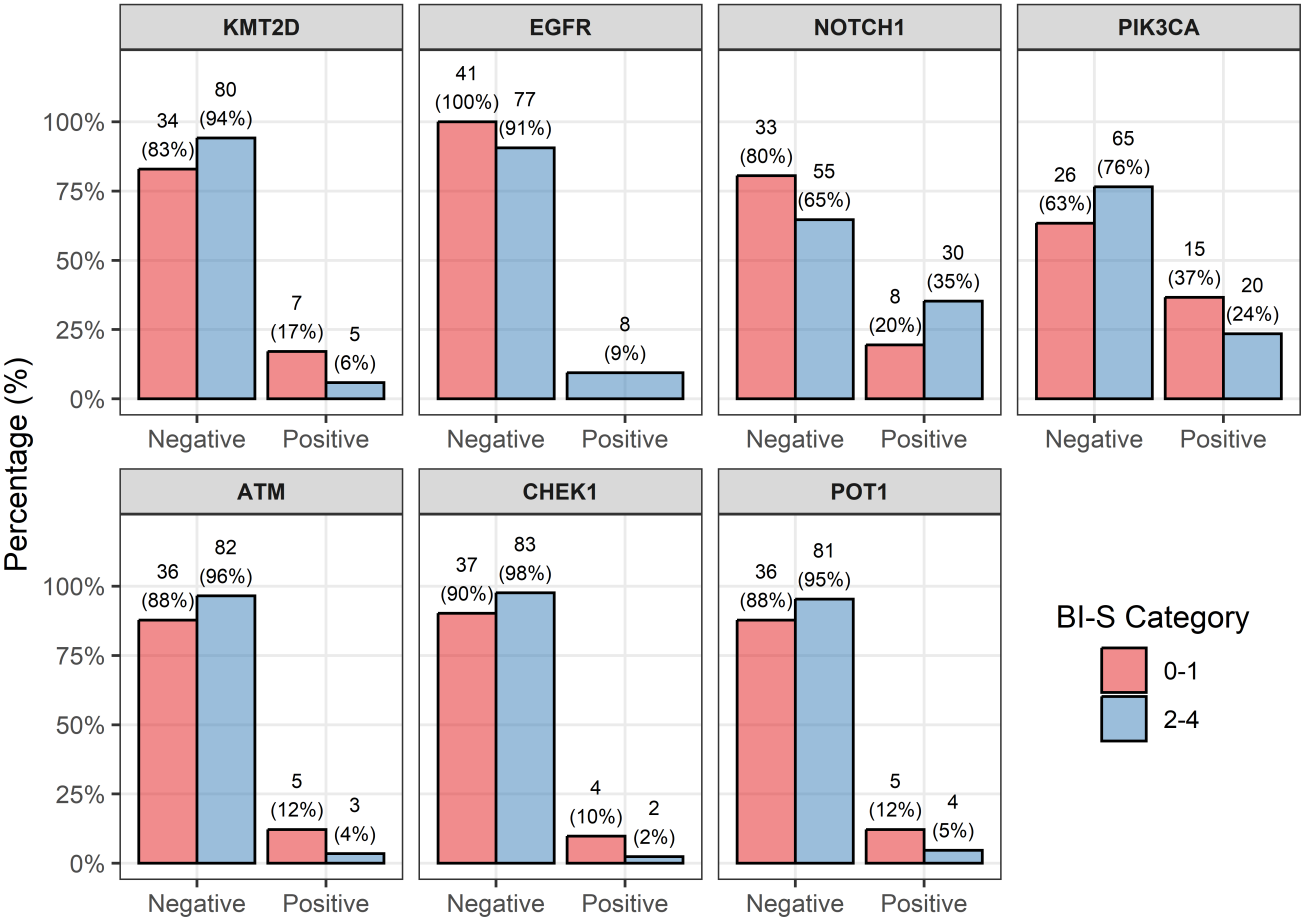
Graphical representation of next‐generation sequencing data in association with B‐IS. The plots depict the distribution of genetic alterations identified in the cohort and their relationship to B‐IS status, focusing on the most frequently altered genes, including *EGFR* amplification, and *KMT2D*, *NOTCH1*, *PIK3CA*, *ATM*, *CHEK1*, and *POT1* mutations. The figure highlights differences in mutation frequency across B‐IS categories, illustrating the mutational landscape in relation to B‐cell infiltration. This figure was created with R software version 4.5.1, package ggpod2 version 4.0.0.

A borderline insignificant association was observed between a low B‐IS and alterations in *KMT2D* (OR = 0.31; 95% CI: 0.07–1.21; *p* = 0.057) and *EGFR* (OR = ∞; 95% CI: 0.86–∞; *p* = 0.053). Insignificant trends towards associations were found between low B‐IS and mutations in *NOTCH1* (OR = 2.24; 95% CI: 0.87–6.33; *p* = 0.10), *PIK3CA* (OR = 0.54; 95% CI: 0.22–1.31; *p* = 0.14), *ATM* (OR = 0.27; 95% CI: 0.04–1.45; *p* = 0.11), *CHEK1* (OR = 0.23; 95% CI: 0.02–1.65; *p* = 0.087), and *POT1* (OR = 0.36; 95% CI: 0.07–1.77; *p* = 0.15). The results are summarized in Table 3 and illustrated in the graphs shown in Figure 6.

**Table 3:** Associations between B‐immunoscore and genomic alterations

Gene	Level	*n*	B‐immunoscore, 0–1, *n* = 41	B‐immunoscore, 2–4, *n* = 85	OR	95% CI		*p*
						Low	High	
*EGFR* amplification	Negative	118	41 (100%)	77 (91%)	∞	0.86	∞	0.053
	Positive	8	0	8 (9%)				
*KMT2D* mutation	Negative	114	34 (83%)	80 (94%)	0.31	0.07	1.21	0.057
	Positive	12	7 (17%)	5 (6%)				
*CHEK1* mutation	Negative	120	37 (90%)	83 (98%)	0.23	0.02	1.65	0.087
	Positive	6	4 (10%)	2 (2%)				
*NOTCH1* mutation	Negative	88	33 (81%)	55 (65%)	2.24	0.87	6.33	0.10
	Positive	38	8 (19%)	30 (35%)				
*ATM* mutation	Negative	118	36 (88%)	82 (97%)	0.27	0.04	1.45	0.11
	Positive	8	5 (12%)	3 (3%)				
*PIK3CA* mutation	Negative	91	26 (63%)	65 (77%)	0.54	0.22	1.31	0.14
	Positive	35	15 (37%)	20 (23%)				
*POT1* mutation	Negative	117	36 (88%)	81 (95%)	0.36	0.07	1.77	0.15
	Positive	9	5 (12%)	4 (5%)				

CI, confidence interval, *p* value in Cox regression; *n*, number; OR, odds ratio.

No associations were detected with the TMB or PD‐L1 expression.

## Discussion

Growing evidence indicates the critical role of tumor‐infiltrating B lymphocytes (TIL‐Bs), consisting of tumor‐infiltrating B cells and plasma cells, as key multifunctional contributors to antitumor immunity. Rather than existing in isolation, TIL‐Bs are typically found in close association with T cells, myeloid cells, and other immune constituents.

The observation that exhausted or dysfunctional CD8^+^ and CD4^+^ T cells within tumors frequently express the B‐cell‐attracting chemokine CXCL13 suggests a programmed mechanism through which T cells recruit B‐cell support in response to persistent tumor antigens. These cellular interactions often lead to the formation of TLSs. Furthermore, TIL‐Bs have been shown to increase the prognostic value of CD4^+^ and CD8^+^ T cells, an effect that is particularly pronounced in tumors containing TLSs [17].

Consequently, tumor‐associated TLSs function as a key site for antigen presentation and the initiation of adaptive immune responses in the tumor microenvironment, and their presence has been correlated with improved prognosis across multiple cancer types [18].

The prognostic value of TLSs has been well documented in various cancers such as head and neck SCC [6], esophageal SCC [7], intrahepatic cholangiocarcinoma [8], and endometrial cancer [19]. TLSs have been consistently associated with favorable clinical outcomes, including improved OS and enhanced responsiveness to immunotherapy.

In contrast, data on the presence and clinical relevance of TLSs in penile SCC remain limited, with a recent study by Tang *et al* being the only one to date reporting a positive prognostic impact and proposing a model linking the presence of TLS to improved outcomes in penile SCC In this study, TLSs were quantified by selecting five random fields within the intra‐ and peritumoral regions [20]. However, our methodology involves a comprehensive enumeration of all TLSs present along the tumor invasion front.

Consistent with previous observations, our study suggests that high TLS number (HR = 2.17; 95% CI: 0.94–5; *p* = 0.069), density (HR = 1.85; 95% CI: 0.93–3.70; *p* = 0.079), and size (HR = 1.59; 95% CI: 0.95–2.70; *p* = 0.079) show insignificant trends towards a more favorable prognosis in pSCC.

Although these associations did not achieve significance at the conventional statistical threshold, the effect sizes and CIs suggest a potentially meaningful biological relationship, which warrants further investigation. The observed relationship between low TLS density and lymphovascular invasion further supports a potential biological relevance of TLS in this context.

Accumulating evidence indicates that TLS formation can be therapeutically induced during cancer treatment interventions [18]. TLSs have also emerged as a promising predictive biomarker for responses to antitumor therapies, including immune checkpoint inhibitors (ICI) and, potentially, chemotherapy [21, 22, 23]. Furthermore, novel nomograms have already been proposed to predict the prognosis of patients with breast cancer brain metastases based on the immune composition of TLSs [24].

Our findings contribute to the growing understanding of the immune microenvironment in pSCC and suggest potential avenues for future investigation of novel therapeutic strategies. While the role of B lymphocytes in the tumor microenvironment is not as well characterized as that of other immune cell types, accumulating evidence indicates that B cells may play a role in antitumor immunity.

Indeed, a diverse array of B‐cell subpopulations has been identified within the TME, spanning from naïve B cells to terminally differentiated plasma cells and memory B cells [9]. For B cells, CD20^+^, CD22^+^, ADAM28^+^, and BIRC5 have been identified within tumor‐associated TLSs and are implicated in enhancing the response to ICI therapy [25, 26, 27]. Their presence within TLSs has been correlated with favorable outcomes in several malignancies, including breast, colorectal, and lung cancers [28, 29, 30].

The prognostic significance of B cells remains uncertain. To date, only a limited number of studies have specifically addressed this question in SCC, with lung SCC being one of the few subtypes investigated [31].

The first study investigating the prognostic impact of B cells and plasma cells in pSCC was conducted by Stenzel *et al* [32], describing their favorable clinical impact. However, studies using tissue microarray techniques are limited by their small sampling area and the risk of missing regions with the highest immune cell infiltration. Whole slide analysis provides a more comprehensive assessment of the tumor immune microenvironment.

To validate the prognostic importance of TLSs and B cells in pSCC, more studies and meta‐analyses are needed. Nevertheless, to date, the overall paucity of studies and heterogeneous methodologies represents a limitation.

With respect to other malignancies, a systematic review by Liu *et al* reported that high levels of TIL‐Bs were associated with favorable OS in lung, esophageal, gastric, colorectal, liver, and breast cancers; improved recurrence‐free survival in lung cancer; and enhanced disease‐free survival in gastrointestinal malignancies [25, 33].

Our results indicate that a low B‐IS (HR = 1.89; 95% CI: 1.18–3.03; *p* = 0.008) correlates significantly with adverse clinical outcomes in pSCC.

The present study has several inherent methodological considerations. The analysis relied on a restricted B‐cell marker panel (CD20, CD138), which allows for a broad assessment of B‐cell infiltration but does not capture the full heterogeneity of B‐cell subpopulations, such as regulatory, memory, or naïve B cells.

The immunoscore approach may represent a possible limitation: Although it represents a pragmatic method in particular studies, a limitation arises when comparing results of numerous studies using variable median‐based thresholds. Furthermore, TLS maturity and organization were not comprehensively assessed, which may, at least in part, reflect the relatively small number of cases with secondary TLSs (*n* = 18) in our cohort.

Future studies incorporating broader immunophenotyping, single‐cell analyses, and larger patient cohorts will be critical to refine cutoff values and elucidate the prognostic and functional roles of distinct B‐cell subsets and TLS features in pSCC.

Recent studies have demonstrated that genomic profiling of HPV‐positive and HPV‐negative pSCC, representing biologically distinct subtypes, can facilitate more precise patient stratification for ICI‐based clinical trials [34]. While ICIs show promise as a potentially effective and tolerable treatment option for a subset of pSCC patients, a more selective, biomarker‐driven approach is essential. For example, the neutrophil‐to‐lymphocyte ratio has emerged as a potential predictive biomarker for assessing ICI response in this patient population [35].

Low B‐IS was associated with *KMT2D* mutation and *EGFR* amplification. Deletion of *KMT2D* has been shown to drive the transformation of lung basal cell organoids into lung SCC. These findings suggest that *KMT2D* loss may sensitize tumors to receptor tyrosine kinase (RTK)‐RAS pathway inhibition, highlighting a potential therapeutic strategy [36]. Other studies suggest its impact on the tumorigenesis of cutaneous SCC, breast cancer, and gastric cancer [37].

The epidermal growth factor receptor (*EGFR*) is a receptor tyrosine kinase that is commonly overexpressed in a variety of malignancies, including SCC. *EGFR* has become a key target for molecularly guided therapies and is currently employed in the treatment of head and neck SCC, oral SCC, and non‐small cell lung SCC [38, 39, 40, 41]. The association with shorter survival in pSCC was established by our group in an earlier study [15]. The exact role of *KMT2D* and *EGFR* in B‐cell antitumor immunity needs to be further elucidated.

Insignificant associations of low B‐IS with alterations in *NOTCH1*, *PIK3CA*, *ATM*, *CHEK1*, and *POT1* have been found. Katarkar *et al* demonstrated that amplification and elevated expression of *NOTCH1* in cancer‐associated fibroblasts in skin SCC may serve as a promising target for stroma‐focused anticancer interventions [42]. *PIK3CA* mutations are more frequently observed in SCCs. These mutations have emerged as promising targets for novel immunotherapeutic strategies, particularly in breast cancer and head and neck SCC, with growing evidence suggesting their potential relevance in oral SCC as well [43, 44, 45, 46].

Recent evidence has demonstrated that specific variants of the *ATM* gene are associated not only with an increased risk of breast cancer development and poorer clinical outcomes, but also with similar associations observed in esophageal SCC and colorectal cancer [47, 48, 49]. The association between shorter survival and predictive factors in penile SCC was also previously delineated by our research group in a prior publication [15].

*CHEK1*, a key effector kinase within the DNA damage checkpoint pathway, demonstrates significant differential expression across multiple cancer types. Notably, reduced *CHEK1* mRNA expression is significantly associated with poor clinical prognosis in patients with gastric and colorectal carcinomas, underscoring its potential utility as a prognostic indicator and therapeutic target [50].

In endometrial cancer, reduced *CHEK1* expression has been identified as a risk factor for disease recurrence and may serve as a stratification marker for patient prognosis and therapeutic decision‐making [51]. As in other SCCs, inhibition of the upregulated ATR–CHEK1 signaling pathway may potentiate the therapeutic efficacy of ionizing radiation in the treatment of oral SCC [52, 53].

While mutations in the Protection of Telomeres 1 (*POT1*) gene are well‐established as factors in the development of melanoma and lung cancer, it has been suggested that *POT1* testing could be beneficial for patients with familial cancer syndromes [54, 55, 56]. To date, there is limited data regarding the role of *POT1* mutations in SCC, except for our own study, which highlights its potential prognostic significance [5]. Again, the exact link between these mutations and B‐cell antitumor immunity remains unclear and has yet to be fully characterized.

The development and implementation of reliable biomarkers to predict the response to immune checkpoint inhibitors in genitourinary malignancies increasingly rely on the characterization of tumor inflammatory signatures, TMB, and PD‐L1 expression [57, 58]. With the increasing integration of NGS technologies into clinical practice, comprehensive genomic profiling is becoming a cornerstone of precision oncology.

This is particularly relevant in pSCC, a disease associated with poor prognosis, where there is a pressing need for refined risk stratification tools. Current therapy for advanced penile cancer is primarily based on cisplatin‐containing chemotherapy regimens, which remain the standard first‐line approach. However, their clinical efficacy is often limited and lacks long‐term durability [57].

In conclusion, while pSCC remains a rare and challenging malignancy, ongoing research into the role of TLSs and B lymphocytes may offer new insights into its pathogenesis and potential therapeutic targets. Our findings indicate that TIL‐Bs and the presence of TLSs in the peritumoral area may hold prognostic significance. However, further validation in larger, independent cohorts as well as investigation of the potential role of modulating the immune microenvironment in this disease is necessary before integration into routine histopathological assessments.

## Author contributions statement

NZ: investigation, methodology, formal analysis, data curation, data analysis, writing, original draft. PW: formal analysis, statistical analysis. MKB: data curation. DČ: data curation. JH: data curation, resources. ZP: data curation. RM: resources, writing, review, and editing. RZ: writing, review, and editing. JH: conceptualization, methodology, data collection, formal analysis, resources, data curation, writing – review and editing, supervision, funding acquisition.

## Supporting information


**Table S1.** Univariate logistic regression/Pearson's chi‐square test for binarized variables

**Table S2.** Complete research data for TLSs

**Table S3.** Complete research data for CD20 and CD138

## Data Availability

The research data are available in the supplementary material, Tables S1–S3.
